# Isolation of human pluripotent stem cell-derived sensory neuron subtypes by immunopanning

**DOI:** 10.3389/fcell.2023.1101423

**Published:** 2023-05-03

**Authors:** Kenyi Saito-Diaz, Christina James, Archie Jayesh Patel, Nadja Zeltner

**Affiliations:** ^1^ Center for Molecular Medicine, University of Georgia, Athens, GA, United States; ^2^ Department of Biochemistry and Molecular Biology, University of Georgia, Athens, GA, United States; ^3^ Department of Cellular Biology, University of Georgia, Athens, GA, United States

**Keywords:** peripheral nervous system (PNS), human pluripotent stem cell (hPSC), sensory neurons, nociceptor, mechanoreceptor, proprioceptor, immunopanning

## Abstract

Sensory neurons (SNs) detect a wide range of information from the body and the environment that is critical for homeostasis. There are three main subtypes of SNs: nociceptors, mechanoreceptors, and proprioceptors, which express different membrane proteins, such as TRKA, TRKB, or TRKC, respectively. Human pluripotent stem cell technology provides an ideal platform to study development and diseases of SNs, however there is not a viable method to isolate individual SN subtype for downstream analysis available. Here, we employ the method immunopanning to isolate each SN subtype. This method is very gentle and allows proper survival after the isolation. We use antibodies against TRKA, TRKB, and TRKC to isolate nociceptors, mechanoreceptors, and proprioceptors, respectively. We show that our cultures are enriched for each subtype and express their respective subtype markers. Furthermore, we show that the immunopanned SNs are electrically active and respond to specific stimuli. Thus, our method can be used to purify viable neuronal subtypes using respective membrane proteins for downstream studies.

## 1 Introduction

The peripheral nervous system (PNS) is necessary to interact with our environment and to maintain body homeostasis (Saito-Diaz and Zeltner, 2019; Jakob et al., 2021). As part of the PNS, sensory neurons (SNs) connect and feed information from organs and tissues to the brain (Abraira and Ginty, 2013; Donnelly, Chen and Ji, 2020). SNs arise from multipotent neural crest (NC) cells that delaminate from the border between the ectoderm and the neural plate during neural tube formation (Marmigère and Ernfors, 2007). NC cells then migrate and cluster in ganglia (such as the dorsal root ganglia, DRG), where they further differentiate into SNs and innervate their target tissues (Marmigère and Ernfors, 2007; Lallemend and Ernfors, 2012). The DRG is composed of three main SN subtypes, which express different cell membrane proteins and detect various stimuli: 1) nociceptors (express TRKA and detect pain and temperature), 2) mechanoreceptors (express TRKB and detect touch), and 3) proprioceptors (express TRKC and detect body position relative to the environment) (Marmigère and Ernfors, 2007). However, during development, expression of different TRKs overlap in some SN subpopulations, for example, in TRKB^+^/C^+^ mechanoreceptors (Lallemend and Ernfors, 2012).

Due to lack of access to relevant tissues, it is difficult to study development and diseases of the PNS, including SNs. The human pluripotent stem cell (hPSC) technology can close this gap. Indeed, many protocols have been established to differentiate hPSCs into SNs, focusing on nociceptors (Pomp et al., 2005; Chambers et al., 2012; Young et al., 2014; Boisvert et al., 2015; Wilson et al., 2018; Holzer et al., 2022), mechanoreceptors (Schrenk-Siemens et al., 2015; Nickolls et al., 2020; Zhu et al., 2021), or proprioceptors (Dionisi et al., 2020). Other protocols can generate all SN subtypes in bulk DRG-like cultures (Alshawaf et al., 2018; Saito-Diaz et al., 2021). Each protocol has certain drawbacks, including the requirement of over expression of exogenous genes (Schrenk-Siemens et al., 2015), or relying on transdifferentiation (Holzer et al., 2022), or the fact that the number of neurons generated is low (Young et al., 2014; Schrenk-Siemens et al., 2015; Alshawaf et al., 2018; Wilson et al., 2018). A common issue is that the cultures are not pure and/or the overall efficiency of SN subtype generation is low. Thus, to further dissect the cellular mechanisms and understand the differences between neuronal subtypes it is necessary to develop methods to isolate and purify SN subtypes. Most current isolation methods rely on fluorescent activated cell sorting (FACS), however this method has drawbacks. While it enables protein or mRNA isolation followed by gene expression analysis (Guez-Barber et al., 2012; Miyazaki et al., 2020), dramatically reduced cell viability post sorting is a big hurdle that disables any further live cell analysis or cell manipulation, including drug testing.

Immunopanning is a method developed to isolate specific cell types and has been used, for example, to isolate astrocytes from murine brains (Zhang et al., 2016; Sloan et al., 2017). It relies on the presence of membrane proteins that can be used to isolate the desired cell types from a heterogenous mixture of cells. Antibodies against the selected protein are coated onto petri dishes (panning dishes). The mixture of detached cells is incubated in the panning dish and the cells expressing the membrane proteins bind to the antibodies. Then the panning dishes are washed to remove unbound cells, and the selected cells are dissociated and further cultured (Zhang et al., 2016; Sloan et al., 2017; Nolle et al., 2021). The gentle nature of the process promotes survival of the panned cells, which makes it an ideal method to isolate neurons for further long-term culture, manipulation, and analysis at various time points.

Here, we describe an updated method of immunopanning to isolate all three SN subtypes differentiated form hPSCs. We used the surface proteins TRKA, TRKB, and TRKC to select for nociceptors, mechanoreceptors, and proprioceptors, respectively. We show that the panned cultures are devoid of other SN subtypes by immunofluorescence and RT-qPCR. Finally, we show that they are electrically active and that they respond only to their expected stimuli. This work updates our previous report (Saito-Diaz et al., 2021) with new conditions to increase yield, purity, and viability of the panned SNs.

## 2 Materials and methods

### 2.1 Reagents

  H9 human embryonic stem cells (WA-09, WiCell)
  Essential 8 (E8) medium (Gibco, cat# A1517001)
  Neurobasal medium (Gibco, cat# 21103-049)
  Essential 6 medium (Gibco, cat# A1516401)
  DMEM medium (Life Technologies, cat# 11965-092)
  SB431542 (R&D Systems, cat# 1614)
  BMP4 (R&D Systems, cat# 314-BP)
  CHIR99021 (R&D Systems, cat# 4423)
  Y-27632 (Biogems, cat# 1293823)
  SU5402 (Biogems, cat# 2159233)
  DAPT (R&D Systems, cat# 2634)
  N2 supplement (Gibco, cat# 17502-048)
  B-27 supplement (Gibco, cat# 12587-010)
  L-glutamine (Thermo, cat# 25030-081)
  GDNF (Peprotech, cat# 450-10)
  BDNF (R&D Systems, cat# 248-BD)
  NGF (Peprotech, cat# 450-01)
  Retinoic acid (Sigma, cat# R2625)
  Vitronectin (VTN, Thermo, cat# A31804)
  Poly-L-ornithine (PO, Sigma, cat# P3655 )
  Fibronectin (FN, Corning, cat# 47743-654)
  Laminin (LM, Cultrex, cat# 3401-010-02)
  BSA (Sigma, cat# A503)
  PBS (Corning, cat# 21-031-CM)
  FBS (Atlanta Biologicals, cat# S11150)
  DNAseI (Roche, cat# 10104159001)
  EDTA (Thermo, cat# AM9262)
  NaCl (Sigma, cat# S5886)
  Accutase (Innovative Cell Technologies, cat# AT-104)
  Earle’s balanced salt solution (EBSS, Sigma, cat# E7510)
  Capsaicin (Sigma, cat# M2028)

### 2.2 Antibodies

  TRKA (R&D Systems, cat# MAB1751R)
  TRKB (R&D Systems, cat# MAB3971)
  TRKB (Alomone labs, cat# ANT-019)
  TRKC (R&D Systems, cat# AF373)
  Anti-mouse IgG (Jackson ImmunoResearch, cat# 115-005-003)
  Anti-goat IgG (Jackson ImmunoResearch, cat# 705-005-003)

Note: the TRKA (cat# MAB1751R), TRKB (cat# MAB3971), and TRKC (cat# AF373) antibodies are used during immunopanning. Their efficiency and suitability for immunopanning is lot dependent, thus they need to be tested carefully.

### 2.3 Oligonucleotides

**Table:** 

Name	Sequence (5′–3′)
BRN3A F	AGT​ACC​CGT​CGC​TGC​ACT​CCA
BRN3A R	TTG​CCC​TGG​GAC​ACG​GCG​ATG
PRPH F	GTG​CCC​GTC​CAT​TCT​TTT​GC
PRPH R	GTC​ACC​ACC​TCC​CCA​TTC​CG
TRKA F	CAC​TAA​CAG​CAC​ATC​TGG​AGA​CC
TRKA R	TGA​GCA​CAA​GGA​GCA​GCG​TAG​A
TRKB F	ACA​GTC​AGC​TCA​AGC​CAG​ACA​C
TRKB R	GTC​CTG​CTC​AGG​ACA​GAG​GTT​A
TRKC F	CCG​ACA​CTG​TGG​TCA​TTG​GCA​T
TRKC R	CAG​TTC​TCG​CTT​CAG​CAC​GAT​G
SST F	CCA​GAC​TCC​GTC​AGT​TTC​TGC​A
SST R	TTC​CAG​GGC​ATC​ATT​CTC​CGT​C
TAC1 F	TTA​CTG​GTC​CGA​CTG​GTA​CGA​C
TAC1 R	CAA​AGA​ACT​GCT​GAG​GCT​TGG​G
KCNK2 F	CTG​CTG​TCC​TGA​GCA​TGA​TTG​G
KCNK2 R	TGT​GAC​GTT​GGC​TGT​CCA​CTC​A
ASIC1 F	GAC​TCC​TAC​AGC​ATC​ACT​GCC​T
ASIC1 R	GCA​CAC​TCC​TTG​TAC​TGC​TCT​G
PIEZO2 F	GAC​GGA​CAC​AAC​TTT​GAG​CCT​G
PIEZO2 R	CTG​GCT​TTG​TTG​GGC​ACT​CAT​TG
SPP1 F	CGA​GGT​GAT​AGT​GTG​GTT​TAT​GG
SPP1 R	GCA​CCA​TTC​AAC​TCC​TCG​CTT​TC
PVALB F	GCT​GAA​CGC​TGA​GGA​CAT​CAA
PVALB R	ACA​TCA​TCC​GCA​CTC​TTT​TTC​TT
GAPDH F	GTC​TCC​TCT​GAC​TTC​AAC​AGC​G
GAPDH R	ACC​ACC​CTG​TTG​CTG​TAG​CCA​A

### 2.4 EDTA dissociation solution

**Table:** 

Reagent	Final concentration	Amount
EDTA	0.5 mM	50 µL
NaCl	30 mM	500 µL
PBS		Up to 50 mL
Total		50 mL

Store at room temperature.

### 2.5 NCC differentiation media 1

Store at 4°C for up to 2 weeks.

**Table:** 

Reagent	Final concentration	Amount
SB431542	10 μM	50 µL
BMP4	1 ng/mL	5 µL
CHIR99021	300 nM	10 mL
Y-27632	10 μM	50 µL
Essential 6 medium		Up to 50 mL
Total		50 mL

### 2.6 NCC differentiation media 2

**Table:** 

Reagent	Final concentration	Amount
SB431542	10 μM	100 µL
CHIR99021	0.75 μM	12.5 µL
SU5402	2.5 μM	6.25 µL
DAPT	2.5 μM	6.25 µL
Essential 6 medium		Up to 100 mL
Total		100 mL

Store at 4°C for up to 2 weeks.

### 2.7 SN differentiation media

**Table:** 

Reagent	Final concentration	Amount
N2 supplement	1X	1 mL
B-27 supplement	1X	2 mL
L-glutamine	2 mM	1 mL
GDNF	20 ng/mL	200 µL
BDNF	20 ng/mL	200 µL
NGF	25 ng/mL	100 µL
Laminin-1	600 ng/mL	60 µL
Fibronectin	600 ng/mL	60 µL
Retinoic acid	0.125 μM	12.5 µL
Neurobasal medium		Up to 100 mL
Total		100 mL

Store at 4°C for up to 2 weeks.

### 2.8 Antibody incubation buffer

**Table:** 

Reagent	Final concentration	Amount
BSA	0.2%	10 µL
DNaseI	1 mg/mL	15 µL
PBS		Up to 5 mL
Total		5 mL

Store at 4°C for up to 2 weeks. Add DNaseI right before using.

### 2.9 Panning buffer

**Table:** 

Reagent	Final concentration	Amount
FBS	20%	1 mL
DNAse	1 mg/mL	15 µL
Y-27632	10 μM	5 µL
PBS		Up to 5 mL
Total		5 mL

Make fresh. Store at 4°C until ready to use.

### 2.10 Dissociation buffer

**Table:** 

Reagent	Final concentration	Amount
Accutase	n.a.	4 mL
EBSS	1X	11 mL
Y-27632	10 µM	15 µL
DNaseI	1 mg/mL	75 µL
Total		15 mL

Make fresh. Keep at room temperature until ready to use.

### 2.11 Dislodge buffer

**Table:** 

Reagent	Final concentration	Amount
FBS	20%	3 mL
Neurobasal medium	40%	6 mL
DMEM medium	40%	6 mL
Y-27632	10 µM	15 µL
DNaseI	1 mg/mL	75 µL
Total		15 mL

Store at 4°C for up to 2 weeks. Add DNaseI and Y-27632 right before using.

### 2.12 VTN plates preparation

1. Mix 120 µL VTN with 12 mL of PBS (for 6-well plates) or 100 µL VTN of 10 mL of PBS (for 10 cm dish).
2. Add 2 mL of the mix to each well of a 6-well plate.
3. Incubate for 1 h at RT.

### 2.13 PO/LM/FN plates preparation

1. Mix 7 mL PBS with 3.5 µL of poly-L-ornithine
2. Add the mix to 10 cm cell culture dish.
3. Incubate at 37°C overnight.
4. The next day, wash the dish 2X with PBS.
5. Mix 7 µL of Laminin-1 and Fibronectin (stock: 1 mg/mL) with 7 mL PBS
6. Add mix to the washed dish.
7. Incubate at 37°C overnight.

## 3 Step by step protocol

### 3.1 Cell culture

1. Grow H9 cells in a 10 cm VTN-coated dish at 37°C in a 5% CO_2_ humidified incubator.
2. Feed cells daily with Essential E8 medium with supplement.
3. When the colonies reach the appropriate size (around day 4) wash the cells with PBS.
4. Add 4 mL of EDTA dissociation solution to the cells.
5. Incubate for 2 min at 37°C.
6. Aspirate the EDTA dissociation solution
7. Resuspend the cells in E8 media and transfer to a new dish at a dilution of 1:10–1:20.

### 3.2 SN differentiation

1. Grow H9 cells in a 10 cm VTN-coated dish until the colonies are ready to split.
2. Wash the cells once with PBS
3. Add 4 mL of EDTA and incubate the dish for 15 min
4. Transfer the cells to a 50 mL conical tube and fill with PBS.
5. Centrifuge tube at 200 × g and resuspend the pellet in 5 mL of NCC Differentiation media 1
6. Count the cells and seed in a 6-well plate coated with VTN at a density of 200,000 cells/cm^2^ in 2 mL/well.
7. The following day (day 1), replace the medium.
8. On day 2, feed the cells with 3 mL of NCC Differentiation media 2.
9. Replace the media every 48 h until day 12.
10. On day 12, wash cells with PBS.
11. Incubate cells with Accutase for 20 min at 37°C.
12. Transfer the cells to a 50 mL conical tube and fill up with PBS.
13. Centrifuge the tube at 200 × g for 4 min at room temperature.
14. Resuspend the cells in SN Differentiation media + 1 μM DAPT + 10 μM Y-27632
15. Seed the cells in a 10 cm PO/LM/FN-coated dish at a density of 250,000 cells/cm^2^.
16. Feed the cells every 2–3 days through day 20.
17. On day 20, remove DAPT and feed the cells until day ∼25.

### 3.3 Immunopanning

1. On day 24, add 13 mL of 50 mM Tris-HCl (pH 9.5) to four 15 mL conical tubes.
2. Add 40 μL of anti-mouse IgG or anti-goat IgG to three tubes from the previous step. Combine 20 μL of anti-mouse IgG and anti-goat IgG (clearing dish) in the remaining tube. Mix all tubes thoroughly.
3. Add the mix from each tube to a petri dish and incubate at 4°C overnight.
4. The next day, wash the dishes three times with PBS.
5. Mix 6.6 μg of TRKA, 10 μg TRKB, or 10 μg TRKC antibody to three tubes containing 5 mL of antibody incubation buffer (one tube per antibody).
6. Add the mix to one petri dish (TRKA and TRKB mix to anti-mouse-IgG-coated dishes, TRKC to anti-goat-IgG-coated dish) and incubate for at least 2 h at room temperature. Add only antibody incubation buffer to the clearing dish.
7. Wash the SNs with PBS.
8. Add 4 mL of Accutase to the cells and incubate for 45 min at 37°C.
9. Resuspend the cells and transfer to a 50 mL conical tube.
10. Fill the tube with PBS and centrifuge it at 200 × g for 4 min.
11. Resuspend the pellet in 5 mL of Panning buffer using a p1000 micropipette.
12. Filter the cells using a 40 μm filter and count them.
13. Wash the clearing dish three times with PBS.
14. Add the cells to the clearing dish and incubate for 10 min at room temperature.
15. Following the incubation, wash the TRKA panning dish three times with PBS.
16. Transfer the SNs from the clearing dish to the TRKA dish and incubate for 15 min at room temperature.
17. Wash the TRKB panning dish three times with PBS.
18. Transfer the SNs from the TRKA panning dish to the TRKB panning dish and incubate for 15 min.
19. Carefully wash the TRKA panning dish (positive selection dish) with PBS 5–7 times. This will remove the unbound cells.
20. Incubate the TRKA dish with 5 mL of dissociation buffer at 37°C for 8 min.
21. Wash the cells off the dish with 10 mL of dislodge buffer.
22. Transfer the cells to a 15 mL conical tube and centrifuge it at 200 × g for 4 min.
23. Resuspend the pellet in 500 μL SN differentiation medium + 10 μM Y-27632.
24. Plate the cells in 96-well plates, or concentrate them in a 10-μL drop and plate in a dried 24-well plate coated with PO/LM/FN. We recommend plating them at a density of at least 100,000 cells/cm^2^.
25. Wash the TRKC panning dish three times with PBS.
26. Transfer the SNs from the TRKB panning dish to the TRKC panning dish and incubate for 15 min.
27. Repeat steps 19 through 24 for the TRKB panning dish.
28. Repeat steps 19 through 24 for the TRKC panning dish.
29. The following day, carefully replace the medium.
30. Replace the medium every 2–3 days.

## 4 Results

**FIGURE 1:**
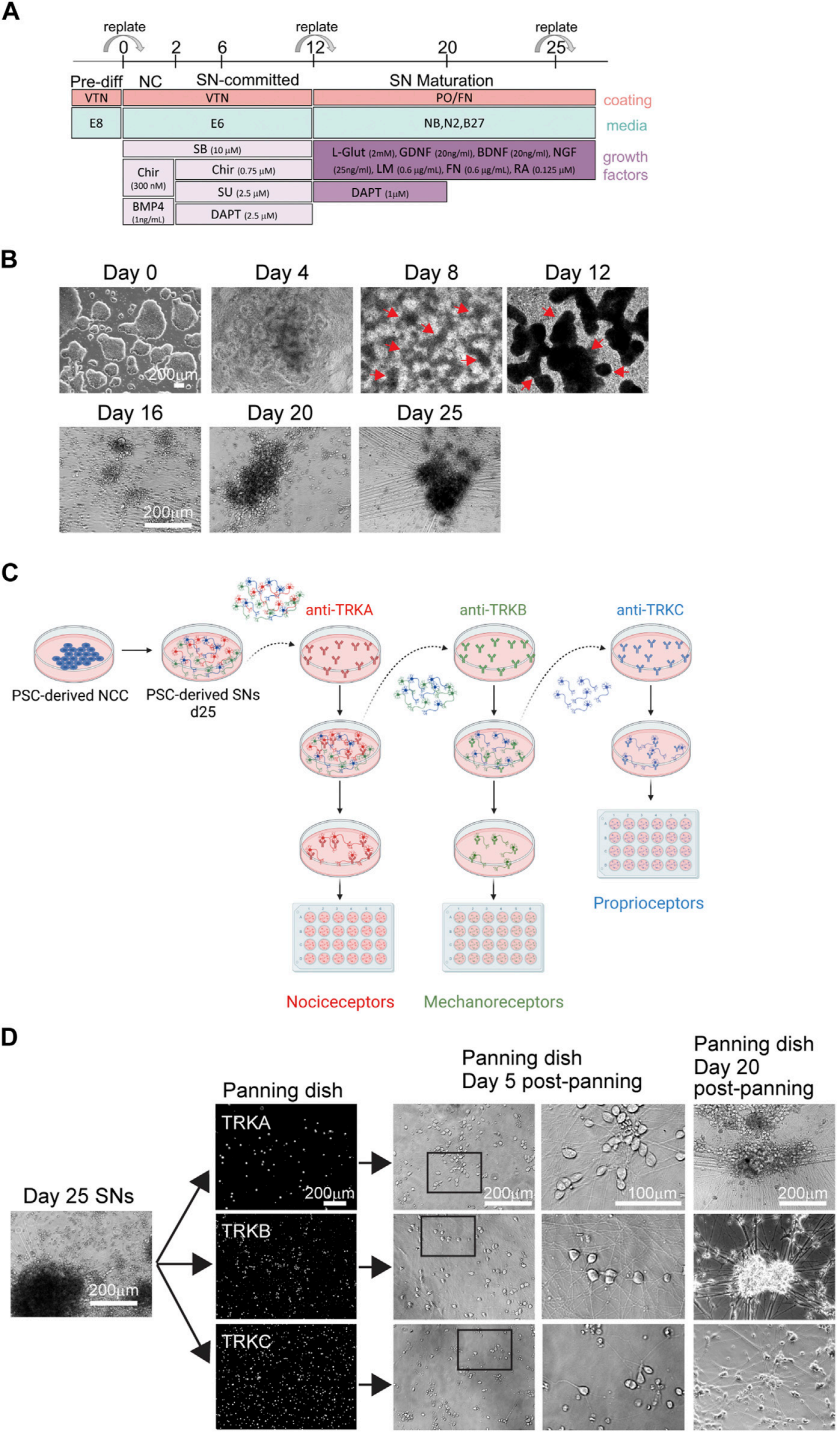
Differentiation of SNs and immunopanning strategy. **(A)** Schematics of SN differentiation. **(B)** Representative brightfield images of hPSC-derived SNs at the pluripotent stage (day 0), NC stage (day 4, 8, and 12), and SN stage (day 16, 20, and 25). Arrows indicate NC cell “ridges.” **(C)** Schematics of the immunopanning technique. **(D)** Representative images of SNs during immunopanning. Day 25 SNs were immunopanned and images were taken at different stages.

We have previously established a protocol to differentiate NC cells and SNs from hPSCs (Saito-Diaz et al., 2021; Saito-Diaz and Zeltner, 2022) (Figure 1A). Starting on day 8, we observe the formation of ridges (i.e., dark clusters) of NC cells (Figure 1B, arrows). Replating these cells on day 12, promotes differentiation into SNs, which are visible on day 16 and by day 20 (Figure 1B). By day 25 the neurons have acquired the morphology characteristic of SNs including axons connecting SN clusters (Figure 1B) (Saito-Diaz et al., 2021). We have shown that these cultures are composed of 70% nociceptors, 30% mechanoreceptors and proprioceptors (Saito-Diaz et al., 2021). Various research applications, including studies on mechanisms of development, axon regeneration, multi-omics, and characterization of neuronal subpopulations can enormously benefit from a method to isolate, healthy SN subtypes. The most common method to isolate and purify live cells is FACS. However, we have experienced that post FACS, sorted neurons do not survive. Similar results were shown in the literature (Guez-Barber et al., 2012; Miyazaki et al., 2020). Thus, more gentle and easy methods are required to isolate such cell types. We have previously shown that these neuron subtypes can be isolated via immunopanning (Saito-Diaz et al., 2021) (Figure 1C). Antibodies against the TRK family of surface proteins, provide an ideal way to isolate individual SN subtypes. TRKA can be used to isolate nociceptors, TRKB is expressed in mechanoreceptors, and TRKC is expressed by proprioceptors (Figure 1C). A subpopulation of SNs express both TRKB and TRKC, which is necessary to take into account when performing the immunopanning. It is important to note that for the success of this procedure the quality of the antibody is of utmost importance. Additionally, it is critical to start with healthy SNs, such as SNs showing clear cell bodies and low number of apoptotic cells (Figure 1D). Resuspended SNs can be observed under the microscope to monitor health of the cells during immunopanning. They should be circular, not forming aggregates and presence of debris should be minimal (Figure 1D). However, as the immunopanning progresses, it is normal to see some debris from dead cells. This does not negatively impact the results. To increase survival, it is critical to add Y- 27632 to the buffers and to the seeding media (SN differentiation medium). Cells that do not attach to the panning dish will move with the media if the dish is gently tapped (Figure 1D). The following day, live SNs are firmly attached to the bottom of the wells, and some are already growing axons. Five days post immunopanning, cells start showing large axon extensions. After 20 days, SNs aggregated and axons are clearly visible by brightfield microscopy (Figure 1D).

**FIGURE 2:**
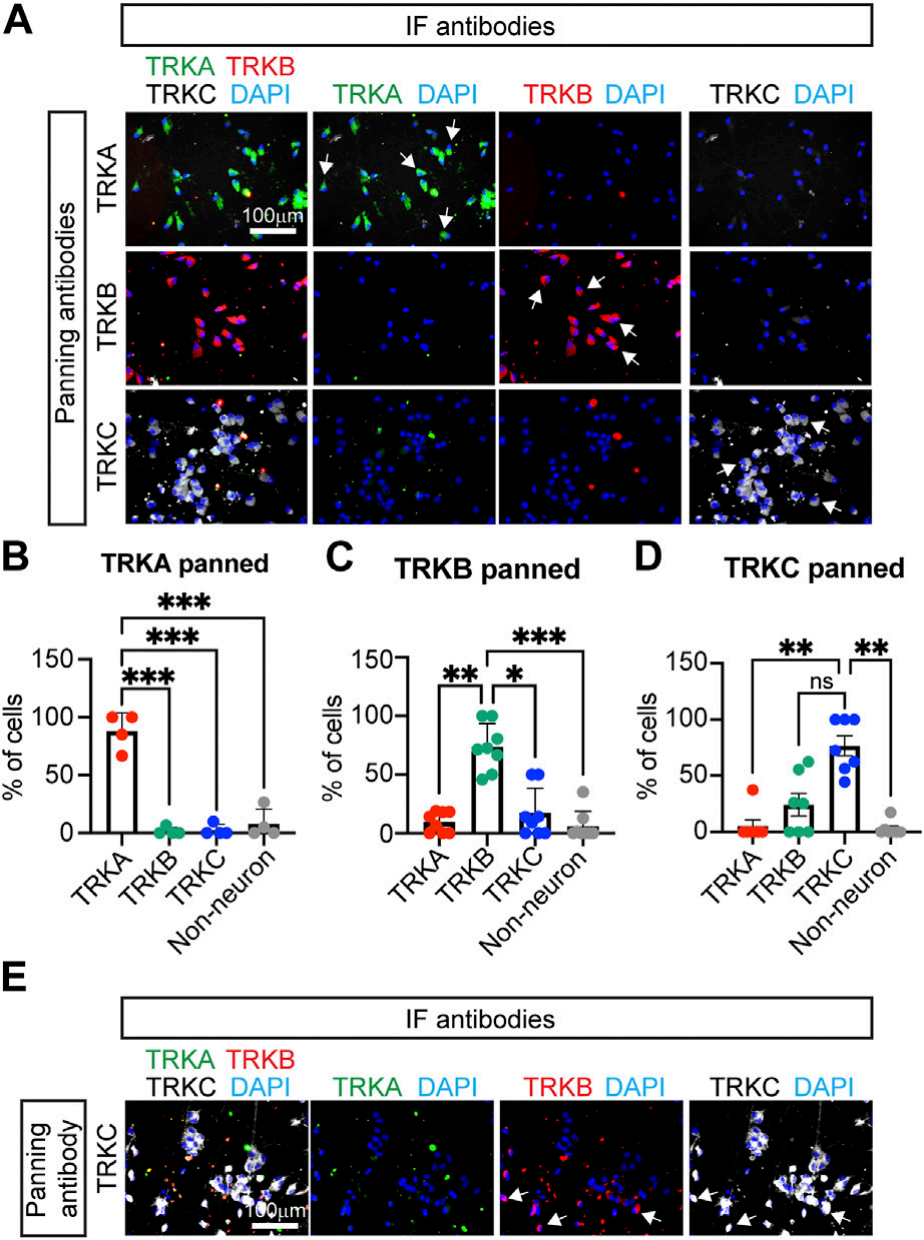
Characterization of immunopanned SNs. **(A)** Representative immunofluorescence images of immunopanned SNs. Cells were fixed 20 days post panning (day 45) and stained for the indicated antibodies. DAPI was used to mark the nucleus. Arrows indicate neurons. **(B–D)** Quantification of IF images from panned SNs using anti-TRKA antibody **(B)**, anti-TRKB **(C)**, and anti-TRKC **(D)** antibodies. Non-neurons are cells that are positive for DAPI but do not express any of the TRK receptors. **(E)** Representative images of TRKC-panned SNs expressing TRKB and TRKC. Arrows indicate TRKB^+^TRKC^+^ neurons. n = 4–8 biological replicates. Graphs show mean ± S.D. One-way ANOVA was used. ns, non-significant, ∗*p* < 0.05, ∗∗*p* < 0.01, ∗∗∗*p* < 0.001.

To confirm that the panned cells indeed had the identity of SNs, we performed IF for the markers TRKA, TRKB, and TRKC. We found that SNs isolated using the TRKA antibody, were negative for TRKB and TRKC (Figure 2A, top row), suggesting that nociceptors were successfully isolated. Additionally, cells that were panned using the TRKB or TRKC antibodies did not express TRKA by IF (Figure 2A and image quantification in Figures 2B–D) suggesting that the correct SN subtypes were successfully isolated. However, some non-neuronal cells were carried over to the final culture, highlighting the importance of the washes after the panning step (Figures 2A–D). We also found presence of TRKB^+^ cells in TRKC-panned SNs. (Figure 2D). This is possible because some TRKB^+^ mechanoreceptors are also positive for TRKC (Figure 2E).

**FIGURE 3:**
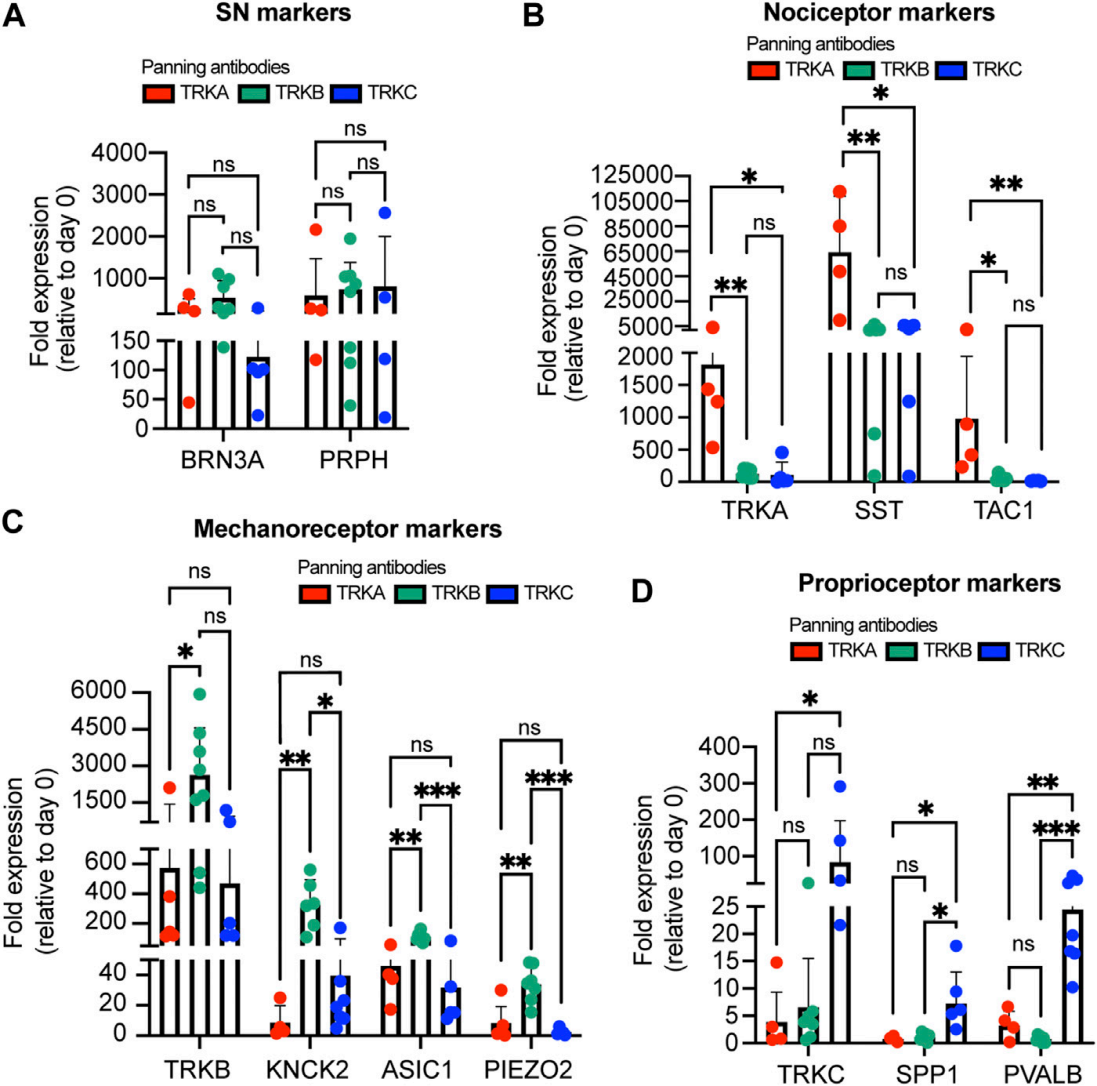
Expression of subtype markers from immunopanned SNs. **(A–D)** SNs were panned on day 25 and the expression of general SN markers **(A)**, nociceptor markers **(B)**, mechanoreceptor markers **(C)**, and proprioceptor markers **(D)** was measured 10 days later by RT-qPCR. *n* = 4–8 biological replicates. Graphs show mean ± S.D. One-way ANOVA was used. ns, non-significant, ∗*p* < 0.05, ∗∗*p* < 0.01, ∗∗∗*p* < 0.001.

To confirm the purity of the panned cultures, we measured expression of additional markers by RT-qPCR. We found all the panned SNs expressed the general SN markers *BRN3A* and *PRPH* (Figure 3A). Agreeing with our previous results, we found that TRKA-panned SNs but not TRKB- or TRKC-panned neurons expressed high levels of nociceptor markers (*TRKA*, *SST*, and *TAC1*) (Figure 3B). Conversely, TRKB-panned SNs showed the highest expression of mechanoreceptor markers (*TRKB*, *KNCK2*, *ASIC1*, *PIEZO2*) (Figure 3C). Interestingly, TRKC-panned SNs also expressed relatively high levels of *TRKB*, possibly coming from cells that are TRKB and TRKC double positive (Figure 3C). Finally, TRKC-panned SNs showed the highest expression of proprioceptor-markers (*TRKC*, *SPP1*, and *PVALB*) (Figure 3D). However, *TRKC* was also highly expressed by TRKB-panned SNs (Figure 3D), possibly due to presence of TRKB^+^/TRKC^+^ SNs. These results suggest that panned cultures consist of mainly the panned SN and few contaminant cells.

**FIGURE 4:**
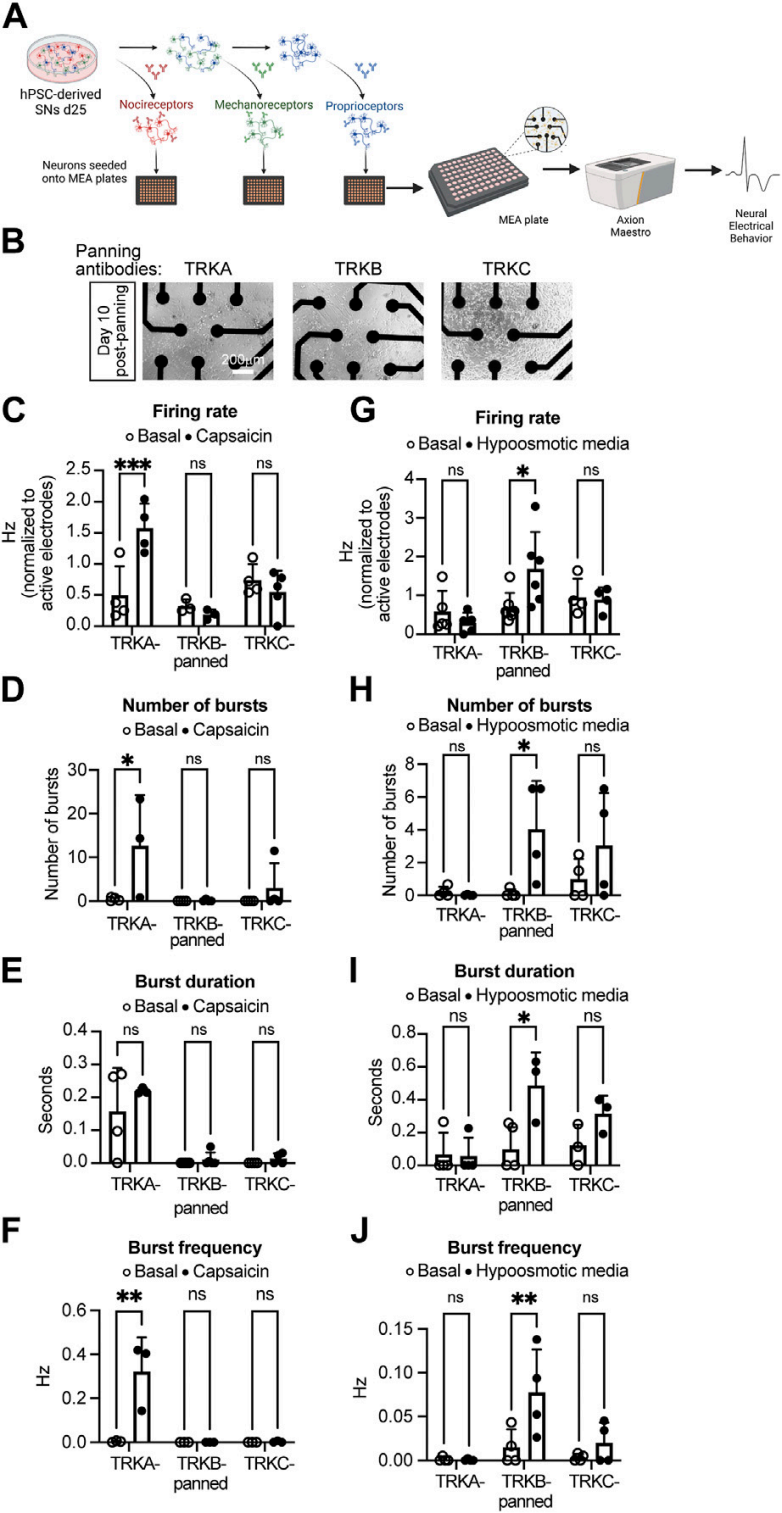
Electrical activity of immunopanned SNs. **(A)** Schematics of the experiment workflow. **(B)** Representative brightfield images of panned SNs seeded in MEA plates (with visible electrodes) 10 days after panning. **(C–F)** Action potential firing rate **(C)**, number of bursts **(D)**, burst duration **(E)**, and burst frequency **(F)** of SNs panned with the indicated antibodies and treated with capsaicin (nociceptor activator). **(G,H)** Action potential firing rate **(G)**, number of bursts **(H)**, burst duration **(I)**, and burst frequency **(J)** of SNs panned with the indicated antibodies and treated with hypoosmotic media (mechanoreceptor activator). *n* = 3–6 biological replicates. Graphs show mean ± S.D. two-way ANOVA was used. ns, non-significant, ∗*p* < 0.05, ∗∗*p* < 0.01, ∗∗∗*p* < 0.001.

To assess whether the panned SNs are still functional, we measured their electrical activity using multielectrode array (MEA) (Figure 4A). After immunopanning, the cells survived and successfully attached to the MEA plates 10 days post panning (Figure 4B). Furthermore, we found that SNs were electrically active at this time point (Figures 4C–J). Additionally, only TRKA-panned SNs were responsive to capsaicin, which is known to activate nociceptors, but not mechanoreceptors or proprioceptors (Figure 4C). Furthermore, capsaicin increased the number and frequency of bursts, but not their duration from TRKA-panned SNs (Figures 4D–F). In contrast, when the panned cells were incubated with hypoosmotic media, which mimics touch by generating pressure on the plasma membrane, only TRKB-panned mechanoreceptors responded appropriately by increasing their firing rate (Figure 4G). This is consistent with increased electrical activity by mechanoreceptors upon touch (Ranade, Syeda and Patapoutian, 2015). Similarly, hypoosmotic media increased the number, duration, and frequency of bursts generated by TRKB-panned SNs (Figures 4H–J). We saw a similar response from TRKC-panned SNs, however the response was lower compared to TRKB-panned cells. Overall, these data suggests that the panned SNs are functionally active and they respond to their proper stimuli.

**FIGURE 5:**
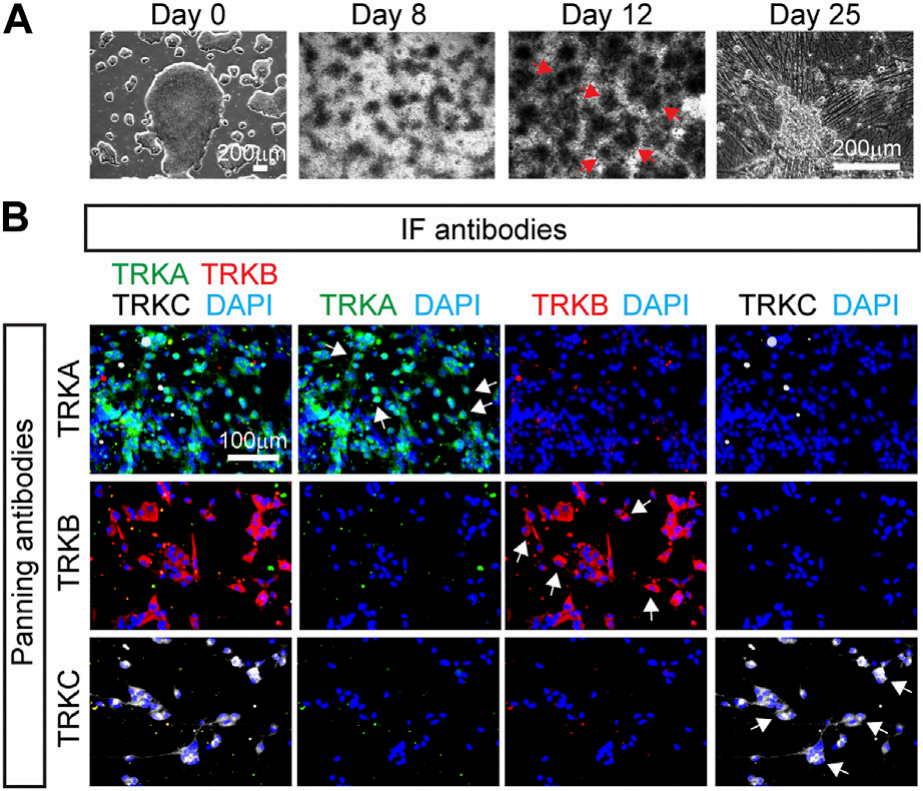
Immunopanning of SNs differentiated from iPSCs. **(A)** Representative brightfield images of SNs differentiated from the iPSC-ctr-C1 line at the pluripotent stage (day 0), NC stage (day 8 and 12), and SN stage (day 25). Arrows indicate NC cell “ridges”. **(B)** Representative immunofluorescence images of immunopanned SNs derived from the iPSC-ctr-C1 line. Cells were fixed 20 days post panning (day 45) and stained for the indicated antibodies.

Finally, we tested whether the immunopanning can be used in other hPSC lines. We differentiated SNs from a previously characterized induced pluripotent stem cells line: iPSC-ctr-C1 (Zeltner et al., 2016; Wu et al., 2022), which underwent the developmental stages previously reported: NC and SN (Figure 5A). We found that SNs from iPSC-ctr-C1 cells can be successfully immunopanned to isolate TRKA^+^, TRKB^+^, and TRKC^+^ SNs (Figure 5B). Thus, these results show that the immunopanning protocol can be successfully applied to a broader range of cells.

## 5 Discussion

Here, we have shown that immunopanning is a simple and straightforward method to isolate live SNs. We used the membrane proteins TRKA, TRKB, and TRKC as markers to isolate nociceptors, mechanoreceptors, and proprioceptors, respectively. However, theoretically, any cell surface marker can be applied to this method. We showed that neurons survive the process healthily, which is a major advance over other methods including FACS, where neurons do not survive well. The panned neurons express markers that are characteristic to each subtype. Furthermore, the neurons are electrically active within 10 days of panning and respond to specific stimuli.

It is important to note that a culture of healthy SNs with large round cell bodies, straight axons protruding from them, with no or minimal presence of apoptotic cells is critical for the success of the protocol. We have immunopanned SNs ranging from day 20–25 and as late as day 40. Interestingly, we found that SNs closer to day 30 tend be more fragile compared to older or younger neurons. However, the reason of this remains unknown. Regardless of their panning day, SNs project axons and fire action potentials around 10 days post panning. Furthermore, regardless of the day the immunopanning was done, our results show that we can enrich the subpopulations of each SN by 2- to 3-fold, compared to the original protocol (Saito-Diaz et al., 2021), highlighting the power of this approach.

The quality and concentration of the antibodies used are also critical for the success of the protocol. We found that variations in lots have a significant impact in the quantity of SNs that can be recovered. However, higher concentration of antibody does not necessarily correlate with increased yield. Thus, antibody titration and careful lot testing is necessary to achieve the highest recovery rate.

The simplicity of our immunopanning method contrasts to other alternatives. For instance, although FACS can be used to also isolate SNs, its application is limited to transcriptomic and proteomic analysis because the harsh protocol results in cell death (Guez-Barber et al., 2012; Miyazaki et al., 2020). A recent protocol (van Niekerk et al., 2022) used MACS columns to isolate live neurons from brain and spinal cord from rats. However, the protocol also resulted in some contaminant cells in the final suspension. Also, the costs of MACS columns and their associated supplies limit their applications. In contrast, our method uses the same principle (positive isolation using antibodies against surface proteins) with reagents and supplies that are commonly found in any research lab and are relatively economical.

There are two major points where our isolation technique can be further improved in the future: 1) the scalability of the protocol and 2) improved purity of cultures. From a 10 cm dish of SNs (with ∼10 × 10^6^ cells) we can isolate up to 2 × 10^6^ cells per subtype. This suggests that there is a large number of neurons that are not captured by the antibodies. A future solution to this might be testing different antibodies and/or using larger panning dishes (Barres, 2014). Such measures may also reduce the number of contaminant cells in each panning dish.

Overall, our immunopanning protocol provides a straightforward, simple, and cost-effective way to isolate live SNs. Isolated neurons can then be further cultured for downstream analysis of live SN. Furthermore, the method we describe can be easily adapted to other neuronal types. The gentle nature of the protocol ensures cell survival; thus we expect that it can be translated to other fields.

## Data Availability

The raw data supporting the conclusion of this article will be made available by the authors, without undue reservation.
